# Lower Urinary Tract Symptoms Are Associated with Increased Risk of Dementia among the Elderly: A Nationwide Study

**DOI:** 10.1155/2015/187819

**Published:** 2015-07-28

**Authors:** Chi-Hsiang Chiang, Ming-Ping Wu, Chung-Han Ho, Shih-Feng Weng, Chien-Cheng Huang, Wan-Ting Hsieh, Ya-Wen Hsu, Ping-Jen Chen

**Affiliations:** ^1^Department of Geriatrics and Gerontology, Chi Mei Medical Center, 901 Zhong-Hua Road, Yongkang District, Tainan 710, Taiwan; ^2^Department of Family Medicine, Chi Mei Medical Center, Tainan 710, Taiwan; ^3^Department of Medical Research, Chi Mei Medical Center, Tainan 710, Taiwan; ^4^Division of Urogynecology, Department of Obstetrics and Gynecology, Chi Mei Medical Center, Tainan 710, Taiwan; ^5^Center of General Education, Chia Nan University of Pharmacy and Science, Tainan 717, Taiwan; ^6^Department of Hospital and Health Care Administration, Chia Nan University of Pharmacy and Science, Tainan 717, Taiwan; ^7^Department of Emergency Medicine, Chi Mei Medical Center, Tainan 710, Taiwan; ^8^Department of Child Care and Education, Southern Taiwan University of Science and Technology, Tainan 710, Taiwan; ^9^Department of Environmental and Occupational Health, College of Medicine, National Cheng Kung University, Tainan 704, Taiwan

## Abstract

Studies show a strong association between dementia and lower urinary tract symptoms (LUTS). The aim of this study was to investigate whether LUTS are a risk factor for cognitive impairment. We enrolled 50-year-old and older subjects with LUTS (LUTS^[+]^) (*n* = 6801) and controls without LUTS (LUTS^[−]^) (*n* = 20,403) from Taiwan's National Health Insurance Research Database. LUTS, dementia, and other confounding factors are defined by International Classification of Diseases, Ninth Revision, Clinical Modification Codes. Participants were recruited from 2000 to 2004 and then followed up until death or the end of 2011. The outcome was the onset of dementia, which was assessed using Poisson regression analysis, Cox hazards models, and Kaplan-Meier survival curves. The incidence of dementia was significantly higher in the LUTS^[+]^ group than in the LUTS^[−]^ group (124.76 versus 77.59/1000 person-years). The increased risk of dementia related to LUTS remained significant after adjustment for potential confounders (adjusted hazard ratio (AHR): 1.61, 95% confidence interval (CI) 1.47–1.76, *P* < 0.0001) and higher than that related to cerebrovascular disease (AHR: 1.43, 95% CI 1.26–1.61, *P* < 0.0001). The outcome suggests the need for early screening and appropriate intervention to help prevent cognitive impairment of patients with LUTS.

## 1. Introduction

Both dementia and lower urinary tract symptoms (LUTS) are common problems in the elderly population, and they have a considerable effect on healthcare and social welfare systems [1, 2]. They increase the caregiver burden [3], dependency, care costs [4, 5], the incidence of skin damage [6], and anxiety and depression, and they reduce quality of life [7, 8].

The incident rate of LUTS is higher in those with dementia than without it [9–11]. Maintaining the continence is a complex mechanism that requires initiating the micturition reflex, responding to the sensation of a full bladder, and inhibiting the passage of urine [12]. Therefore, the progressive impairment of global brain function may have a substantial influence on the micturition control [7, 13].

Grant et al., however, found that the median interval between the onset of dementia and LUTS is only 9–11 months [9]. The clinical presentation implied that the functional decline in cognition and micturition may be concomitant. Moreover, recent researches demonstrated that LUTS may facilitate the progress of cardiovascular insults through multifactorial mechanism such as autonomic nervous dysfunction, affective disorders, or the adverse effects of drugs [14], and LUTS were identified as a significant predictor of acute cardiovascular events [15, 16]. Cardiovascular disease is a known risk factor of symptomatic or healthcare-seeking dementia [17, 18]. It is unclear whether LUTS are an early prodrome of declining global brain function or a risk factor of cognitive impairment.

We used Taiwan's National Health Insurance Research Database (NHIRD) for a cohort study to confirm the hypothesis that LUTS are associated with a higher risk of dementia.

## 2. Materials and Methods

### 2.1. Data Source

Our data source is a randomly sampled cohort of 1 million people from the general population in NHIRD from 2000 to 2011. It is a representative cohort of Taiwan's population of 23 million. More than 99% of the citizens are the beneficiaries covered by National Health Insurance (NHI) for their medical expenditures. All the claim data of the healthcare services, including hospitalization and outpatient clinics, has been collected and encrypted in the NHIRD. Personal information in this dataset cannot be identified after encryption, and using the data must be authorized by the Taiwan Bureau of National Health Insurance (BNHI). Confidentiality assurances are addressed by following the data regulations of the BNHI. Our research protocol was approved by the institutional review board of Chi-Mei Medical Center.

### 2.2. Definition of LUTS

We identified patients who used outpatient services or who made hospitalization claims during the study period with the following categories of The International Classification of Diseases, Ninth Revision, Clinical Modification (ICD-9-CM) Codes: (a) storage symptoms, including hypertonicity of the bladder (ICD-9-CM code 596.51), stress urinary incontinence in women (625.6) and men (788.32), urgent incontinence (788.31), frequency and polyuria (788.4), nocturnal enuresis (788.36), nocturia (788.43), and mixed incontinence (788.33); (b) voiding symptoms, including retention of urine (788.2), splitting and slowing of urine stream (788.6), and post-void dribbling (788.35) [19]. We excluded the codes of benign prostatic hyperplasia that seem to be a kind of structural obstruction of urine flow outlet rather than nervous system degeneration.

### 2.3. Identifying Patients

We defined the recruitment period of 2000 to 2004 and identified patients with either of the following criteria as the LUTS group: (1) at least three outpatient service claims with the codes of LUTS at any clinics within one year after the first LUTS code; (2) any one single hospitalization with LUTS among the 5 principal claims diagnosis codes [19]. The date of the first LUTS code for every patient was designated as the index date of entry. We excluded those patients with any one time ICD-9-CM codes of dementia (290.0–290.4, 331.0, 331.82) before the index entry date. From patients without LUTS and free of dementia, a comparison group was assembled by matching LUTS patients with 3 non-LUTS patients on age and gender. The comorbidities of interest were diabetes mellitus (DM, ICD-9-CM code 250), hyperlipidemia (272), hypertension (HTN) (401–405), ischemic heart disease/coronary artery disease (CAD) (410–414), cerebrovascular disease (430–438), and atrial fibrillation (AF) (427.31).

### 2.4. Follow-Up and Outcome Measures

The major outcome of this study was the first diagnosis of dementia: senile and presenile dementia (ICD-9-CM codes 290.0-3), vascular dementia (290.4), Alzheimer's disease (331.0), and dementia with Lewy bodies/Parkinsonism with dementia (331.82). The diagnosis of dementia had to fit either of the following criteria: (1) at least three outpatient service claims with the codes of dementia at any clinics within one year after the first dementia code and (2) any one single hospitalization with dementia among the 5 principal claims diagnosis codes. The identified patients were followed up until death or the end of 2011.

### 2.5. Statistical Analysis

Pearson's *χ* ^2^ test was used to estimate the differences in age group, gender, selected comorbidities (HTN, DM, CAD, hyperlipidemia, cerebrovascular disease, and AF), and dementia between the two cohorts. The difference in mean age between the LUTS and the non-LUTS groups was determined using the Student's *t*-test.

The dementia incidence rate was calculated by dividing the number of dementia patients by the total person years of both groups. Absolute risk estimates were calculated as rates per 10,000 person-years of observation. A Poisson regression with total person-years as an offset variable was used to calculate the incidence rate ratios of dementia. In addition, Kaplan-Meier curves and the log-rank test were used to describe the proportion of dementia-free patients and compare risk difference between the LUTS and the non-LUTS groups. Cox regression analysis was used to obtain adjusted hazard ratios (AHR) for potential confounding. SAS 9.3 (SAS Institute, Cary, NC) was used for all statistical analyses. Significance was set at *P* < 0.05. Kaplan-Meier curves were plotted using STATA 12 (Stata Corp.* College Station*, TX).

## 3. Results

We enrolled 6801 patients with LUTS in the LUTS^[+]^ study group and 20,403 patients in the LUTS^[−]^ control group. Age (mean: 67.29 ± 9.42 years) and gender distributions were comparable. The LUTS^[+]^ group had a significantly higher prevalence of HTN, DM, CAD, hyperlipidemia (marginally significant), AF, and cerebrovascular disease, which were known as comorbidities of LUTS in other studies [14, 15, 20]. These factors were adjusted for affecting reason. Significantly (*P* < 0.0001) more LUTS^[+]^ group patients (741 (10.9%)) than LUTS^[−]^ group patients (1418 (6.9%)) had dementia during the follow-up. The median time span from the enrollment to the onset of dementia was significantly (*P* = 0.0002) shorter in the LUTS^[+]^ group than in the LUTS^[−]^ group (Table 1).

**Figure 1:**
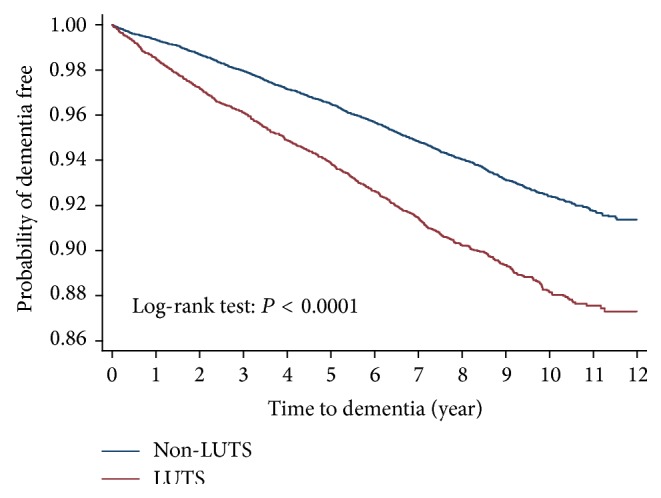
Kaplan-Meier plot for patients with dementia by lower urinary tract symptoms (LUTS).

The incidence rate ratio of dementia in the LUTS^[+]^ group to the LUTS^[−]^ group is 1.63 (95% confidence interval (CI) 1.49–1.78, *P* < 0.0001) after adjusting age and gender (Table 2). With the increase of age, the incidence rate of dementia rose as well, while the risk ratio of LUTS^[+]^ group to the LUTS^[−]^ group showed a reverse *J* curve with the peak risk ratio of 2.0 in the 60–70-year-old subgroup. LUTS had a consistent impact on higher risk of dementia in each subgroup stratified by different cardiovascular confounding factors. Kaplan-Meier survival curves showed that the patients in the LUTS^[−]^ group were less likely to have dementia than those in the LUTS^[+]^ group (Figure 1).

In a Cox regression model, age remained the most critical factor for the onset of dementia. CAD, hyperlipidemia, and AF had no significant impact on the risk of dementia after adjusting for all other confounders. LUTS were significantly associated with a comparatively greater risk of subsequent dementia (AHR = 1.61, 95% CI 1.47–1.76, *P* < 0.0001) compared to other vascular risk factors including cerebrovascular disease (AHR = 1.43, 95% CI 1.26–1.61, *P* < 0.0001) (Table 3).

## 4. Discussion

In this nationwide population-based cohort study using data from Taiwan's NHIRD, LUTS were identified as a risk factor of dementia after adjusting for multiple comorbidities. It is noteworthy that the AHR of dementia in LUTS^[+]^ patients (1.61) was significantly higher than that of cerebrovascular disease in LUTS^[+]^ patients (1.43). To our knowledge, the finding that LUTS might be a prodrome of cognitive deficiency or a risk factor of dementia has seldom been mentioned in other research. Using a large nationwide population-based sample might give the finding persuasive statistical power.

The mechanism of micturition involves the cerebral cortex, the pons, the spinal cord, the autonomic and somatic nervous systems, the sensory system of the lower urinary tract, and the anatomical structure of the lower urinary tract itself [21]. Therefore, LUTS would occur if there was any error in the mechanism of micturition. Degeneration of global lobes, white matter lesions, and microvascular brain disease have been associated with LUTS [11, 12, 22]. Sakakibara et al. demonstrated that, in people with varying degrees of leukoaraiosis, LUTS might precede occurrence of cognitive disorder [23]. They also concluded that small vessel disease of the brain can cause incontinence, which maybe the initial manifestation of dementia in some patients [24]. It appeared that LUTS might be the early clinical symptom of the observed white matter changes and microvascular brain disease.

LUTS were associated with a higher vascular risk in other research [16, 25]. Lin et al. found that LUTS remained a significant predictor for acute cardiovascular events, mainly stroke [15]. Kotsoris et al. reported that urinary disturbances predated overt multi-infarct dementia by more than 5 years in up to 50% of patients [26]. This implied that LUTS may cause the decline of global brain function by increasing the rate of vascular injury. We found, however, a significantly higher risk of dementia in the LUTS^[+]^ group after adjustment for confounding effects by cerebrovascular disease and other cardiovascular risk factors. Thus, the role of LUTS in the pathogenesis of dementia might be complex.

Disorders of autonomic nervous system are also associated with LUTS [27, 28]. The most common example is diabetes, which causes LUTS by autonomic neuropathy and is also involved in the decline of cognitive function [29]. The central control for the autonomic system includes the insular cortex, amygdala, hypothalamus, periaqueductal gray, parabrachial nucleus, nucleus of the solitary tract, and medulla [30]. Several components of the central autonomic networks are affected and characterized by the presence of intracellular inclusions containing *α*-synuclein in neurodegenerative disorders, such as multiple system atrophy and Lewy body disorders [30, 31]. Braak proposed that an immunocytochemical analysis might indicate preclinical neuropathological degeneration of autonomic control in the insular cortex and the brainstem before the onset of obvious cognitive dysfunction in individuals with Alzheimer's disease [32]. Collins et al. also reported that participants with mild cognitive impairment were about 6 times more likely than controls without cognitive impairment to have autonomic dysfunction [33]. LUTS, which result from autonomic dysfunction, may therefore be indicative of early stage of cognition decline and consequent dementia.

Pharmacological treatment for LUTS involves using antimuscarinic or anticholinergic agents, which have adverse effects on cognitive function [34, 35]. The change in cognitive function is caused by the drugs' ability to cross the blood-brain barrier and block muscarinic and cholinergic receptors in the central nervous system (CNS). Results from electroencephalographic data suggest that oxybutynin has a greater effect on central nervous system than trospium and tolterodine [36]. The elderly patients who take these antimuscarinic and anticholinergic agents as therapy for LUTS may have a higher risk of dementia.

LUTS negatively affect quality of life and cause depression, anxiety, and social withdrawal [7, 8, 37]. Thor et al. concluded that the monoamine neurotransmitters serotonin (5-HT) and noradrenaline (NA) are involved in the physiological processes of LUTS and major depressive disorder [37]. Mild worry symptoms and anxiety can predict declines in learning and memory [38, 39]. Geda et al. also suggested that agitation, anxiety, irritability, and depression increased the risk for later cognitive impairment [40]. For this reason, LUTS may increase the risk of cognitive dysfunction by causing neuropsychiatric disorders.

The main strength of this cohort study is the large size of Taiwan's nationwide NHIRD random sample from the general population. However, our study had some limitations. First, the lack of data from the population in low socioeconomic status without continuous health insurance coverage may lead to the under-ascertainment of dementia. However, the steady and high percentage of citizens coverage (>98% since 2002 and 99.51% in 2011) [41] by NHI made the underestimation much less marked. Second, we could neither classify the clinical severity or manifestations of LUTS and dementia nor demonstrate the linkage between subtypes of LUTS and dementia. Third, we lacked data about drugs and psychosocial connection which may have affected both LUTS and cognition. Thus, additional research is needed to identify the mechanism that led to the results in our study.

In conclusion, LUTS increase the risk of dementia in the aging population. To prevent or reduce the predisposing factors of cognitive impairment, early screening and timely intervention, such as nonpharmacological therapy for the elderly patients with LUTS, should be considered. Additional investigations are necessary to evaluate the association of the clinical severity of disease, the connection between different types of LUTS and dementia, the effect of pharmacological therapy, and the psychosocial connection between LUTS and cognitive dysfunction.

## Figures and Tables

**Table 1:** Demographic information.

	Non-LUTS (*n* = 20403)	LUTS (*n* = 6801)	*P* ^*^
Age (years) (mean ± SD)	67.29 ± 9.42	67.29 ± 9.42	0.9994
Age (years)			
50~60	5171 (25.34)	1723 (25.33)	1.0000
60~70	6491 (31.81)	2164 (31.82)	
70~80	7071 (34.66)	2356 (34.64)	
≥80	1670 (8.19)	558 (8.20)	
Gender			
Male	11469 (56.21)	3823 (56.21)	1.0000
Female	8934 (43.79)	2978 (43.79)	
Hypertension			
Yes	6949 (34.06)	2420 (35.58)	0.0220
No	13454 (65.94)	4381 (64.42)	
Diabetes			
Yes	2985 (14.63)	1141 (16.78)	<0.0001
No	17418 (85.37)	5660 (83.22)	
Coronary artery disease			
Yes	2408 (11.80)	907 (13.34)	0.0008
No	17995 (88.20)	5894 (86.66)	
Hyperlipidemia			
Yes	1576 (7.72)	574 (8.44)	0.0582
No	18827 (92.28)	6227 (91.56)	
Cerebrovascular disease			
Yes	1591 (7.80)	707 (10.40)	<0.0001
No	18812 (92.20)	6094 (89.60)	
Atrial fibrillation			
Yes	229 (1.12)	101 (1.49)	0.0180
No	20174 (98.88)	6700 (98.51)	
Time to dementia (year)			
Median (IQR)	4.97 (2.57–7.07)	4.37 (1.93–6.61)	0.0002
Dementia			
Yes	1418 (6.95)	741 (10.90)	<0.0001
No	18985 (93.05)	6060 (89.10)	

^*^It is determined using Student's *t*-test or the Wilcoxon test for continuous variables and the *χ* ^2^ test for categorical variables. Data are number (%). LUTS: lower urinary tract symptoms; SD: standard deviation; IQR: interquartile range.

**Table 2:** Incidence of dementia in LUTS patients.

	Non-LUTS (*n* = 20403)				LUTS (*n* = 6801)				Adjusted IRR^*^ (95% CI)	*P*
	*N*	Dementia number	PY	IR	*N*	Dementia number	PY	IR		
Total	**20403**	**1418**	**182760.38**	**77.59**	**6801**	**741**	**59392.21**	**124.76**	**1.63 (1.49–1.78)**	**<0.0001**
Age (years)										
50~60	5171	67	47695.02	14.05	1723	36	15834.09	22.74	1.62 (1.08–2.43)	0.0198
60~70	6491	284	59208.05	47.97	2164	185	19289.33	95.91	2.00 (1.66–2.41)	<0.0001
70~80	7071	804	61574.10	130.57	2356	396	19740.50	200.60	1.54 (1.37–1.74)	<0.0001
≥80	1670	263	14283.21	184.13	558	124	4528.29	273.83	1.49 (1.20–1.84)	<0.0003
Gender										
Male	11469	814	101731.17	80.02	3823	403	33165.82	121.51	1.52 (1.36–1.72)	<0.0001
Female	8934	604	81029.20	74.54	2978	338	26226.39	128.88	1.76 (1.54–2.01)	<0.0001
Hypertension										
No	13454	809	121784.75	66.43	4381	393	39480.85	99.54	1.49 (1.32–1.69)	<0.0001
Yes	6949	609	60975.62	99.88	2420	348	19911.36	174.78	1.81 (1.59–2.06)	<0.0001
Diabetes										
No	17418	1156	156414.36	73.91	5660	585	49944.80	117.13	1.62 (1.47–1.79)	<0.0001
Yes	2985	262	26346.02	99.45	1141	156	9447.41	165.13	1.62 (1.33–1.98)	<0.0001
Coronary artery disease										
No	17995	1180	161652.69	73.00	5894	614	51908.31	118.29	1.66 (1.50–1.83)	<0.0001
Yes	2408	238	21107.69	112.76	907	127	7483.90	169.70	1.48 (1.19–1.84)	0.0004
Hyperlipidemia										
No	18827	1311	169048.45	77.55	6227	665	54693.83	121.59	1.58 (1.44–1.74)	<0.0001
Yes	1576	107	13711.93	78.03	574	76	4698.38	161.76	2.16 (1.61–2.91)	<0.0001
Cerebrovascular disease										
No	18812	1220	169158.45	72.12	6094	607	53732.30	112.97	1.59 (1.45–1.76)	<0.0001
Yes	1591	198	13601.92	145.57	707	134	5659.92	236.75	1.66 (1.33–2.07)	<0.0001
Atrial fibrillation										
No	20174	1395	180772.73	77.17	6700	719	58568.11	122.76	1.61 (1.47–1.76)	<0.0001
Yes	229	23	1987.65	115.72	101	22	824.10	266.96	2.27 (1.26–4.06)	0.0061

CI: confidence interval; IR: incidence rate, per 1000 person-years; IRR: incidence rate ratio; LUTS: lower urinary tract symptoms; PY: person-years.

^*^The IRR was adjusted by age and gender.

**Table 3:** Cox proportional hazards regression analyses for the risk of dementia stratified by confounding factors.

	Crude hazards (95% CI)	*P*	Adjusted hazards^*^ (95% CI)	*P*
LUTS				
No	1.00 (ref.)		1.00 (ref.)	
Yes	1.61 (1.47–1.76)	<0.0001	1.61 (1.47–1.76)	<0.0001
Age (years)				
50~60	1.00 (ref.)		1.00 (ref.)	
60~70	3.69 (2.98–4.56)	<0.0001	3.65 (2.94–4.52)	<0.0001
70~80	9.11 (7.45–11.14)	<0.0001	9.02 (7.36–11.07)	<0.0001
≥80	12.71 (10.23–15.80)	<0.0001	12.40 (9.94–15.44)	<0.0001
Gender				
Male	1.00 (ref.)		1.00 (ref.)	
Female	0.97 (0.90–1.06)	0.5509	1.30 (1.19–1.41)	<0.0001
Hypertension				
No	1.00 (ref.)		1.00 (ref.)	
Yes	1.59 (1.46–1.73)	<0.0001	1.12 (1.02–1.23)	0.0136
Diabetes				
No	1.00 (ref.)		1.00 (ref.)	
Yes	1.38 (1.24–1.54)	<0.0001	1.13 (1.01–1.27)	0.0286
Coronary artery disease				
No	1.00 (ref.)		1.00 (ref.)	
Yes	1.52 (1.36–1.70)	<0.0001	1.03 (0.92–1.16)	0.5793
Hyperlipidemia				
No	1.00 (ref.)		1.00 (ref.)	
Yes	1.12 (0.97–1.31)	0.1304	1.01 (0.86–1.18)	0.9046
Cerebrovascular disease				
No	1.00 (ref.)		1.00 (ref.)	
Yes	2.10 (1.87–2.36)	<0.0001	1.43 (1.26–1.61)	<0.0001
Atrial fibrillation				
No	1.00 (ref.)		1.00 (ref.)	
Yes	1.81 (1.35–2.44)	<0.0001	1.07 (0.80–1.45)	0.6457

^*^The model was adjusted by the variables which were listed above.

CI: confidence interval; LUTS: lower urinary tract symptoms; ref.: reference.

## References

[1] Wagg A., Gibson W., Johnson T. (2014). Urinary incontinence in frail elderly persons: report from the 5th international consultation on incontinence. *Neurourology and Urodynamics*.

[2] Lawhorne L. W., Ouslander J. G., Parmelee P. A., Resnick B., Calabrese B. (2008). Urinary incontinence: a neglected geriatric syndrome in nursing facilities. *Journal of the American Medical Directors Association*.

[3] Kamiya M., Sakurai T., Ogama N., Maki Y., Toba K. (2014). Factors associated with increased caregivers' burden in several cognitive stages of Alzheimer's disease. *Geriatrics and Gerontology International*.

[4] Talley K. M. C., Wyman J. F., Bronas U. G., Olson-Kellogg B. J., McCarthy T. C., Zhao H. (2014). Factors associated with toileting disability in older adults without dementia living in residential care facilities. *Nursing Research*.

[5] Heinen I., van den Bussche H., Koller D. (2014). Morbidity differences according to nursing stage and nursing setting in long-term care patients: results of a claims data based study. *Zeitschrift für Gerontologie und Geriatrie*.

[6] Rolnick S., Bliss D. Z., Jackson J. M., Arntson C., Mullins J., Hepburn K. (2013). Healthcare providers' perspectives on communicating incontinence and skin damage information with patients with dementia and their family caregivers: a descriptive study. *Ostomy Wound Management*.

[7] Miu D. K., Lau S., Szeto S. S. (2010). Etiology and predictors of urinary incontinence and its effect on quality of life. *Geriatrics and Gerontology International*.

[8] Coyne K. S., Wein A. J., Tubaro A. (2009). The burden of lower urinary tract symptoms: evaluating the effect of LUTS on health-related quality of life, anxiety and depression. *BJU International*.

[9] Grant R. L., Drennan V. M., Rait G., Petersen I., Iliffe S. (2013). First diagnosis and management of incontinence in older people with and without dementia in primary care: a cohort study using the health improvement network primary care database. *PLoS Medicine*.

[10] Skelly J., Flint A. J. (1995). Urinary incontinence associated with dementia. *Journal of the American Geriatrics Society*.

[11] Sakakibara R., Uchiyama T., Yamanishi T., Hattori T. (2004). Urinary function in patients with corticobasal degeneration; comparison with normal subjects. *Neurourology and Urodynamics*.

[12] Jirovec M., Altman H. (1987). Urine control in patients with chronic degenerative brain disease. *Alzheimer’s Disease*.

[13] Lee C.-Y., Chen L.-K., Lo Y.-K. (2011). Urinary incontinence: an under-recognized risk factor for falls among elderly dementia patients. *Neurourology and Urodynamics*.

[14] Asche C. V., Kim J., Kulkarni A. S., Chakravarti P., Andersson K.-E. (2012). Presence of central nervous system, cardiovascular and overall co-morbidity burden in patients with overactive bladder disorder in a real-world setting. *BJU International*.

[15] Lin H.-J., Weng S.-F., Yang C.-M., Wu M.-P. (2013). Risk of hospitalization for acute cardiovascular events among subjects with lower urinary tract symptoms: a nationwide population-based study. *PLoS ONE*.

[16] Parthasarathy S., Fitzgerald M., Goodwin J. L., Unruh M., Guerra S., Quan S. F. (2012). Nocturia, sleep-disordered breathing, and cardiovascular morbidity in a community-based cohort. *PLoS ONE*.

[17] Whitmer R. A., Sidney S., Selby J., Claiborne Johnston S., Yaffe K. (2005). Midlife cardiovascular risk factors and risk of dementia in late life. *Neurology*.

[18] Newman A. B., Fitzpatrick A. L., Lopez O. (2005). Dementia and Alzheimer's disease incidence in relationship to cardiovascular disease in the cardiovascular health study cohort. *Journal of the American Geriatrics Society*.

[19] Wu M.-P., Hsu Y.-W., Weng S.-F., Ho C.-H., Wang J.-J., Tong Y.-C. (2013). Healthcare-seeking prevalence of lower urinary tract symptoms among national health insurance enrollees in Taiwan, 2000–2009. *Urology*.

[20] Moul S., McVary K. T. (2010). Lower urinary tract symptoms, obesity and the metabolic syndrome. *Current Opinion in Urology*.

[21] Ouslander J. G. (2004). Management of overactive bladder. *The New England Journal of Medicine*.

[22] Perneczky R., Diehl-Schmid J., Förstl H., Drzezga A., May F., Kurz A. (2008). Urinary incontinence and its functional anatomy in frontotemporal lobar degenerations. *European Journal of Nuclear Medicine and Molecular Imaging*.

[23] Sakakibara R., Hattori T., Uchiyama T., Yamanishi T. (1999). Urinary function in elderly people with and without leukoaraiosis: relation to cognitive and gait function. *Journal of Neurology Neurosurgery and Psychiatry*.

[24] Sakakibara R., Panicker J., Fowler C. J. (2012). Vascular incontinence: incontinence in the elderly due to ischemic white matter changes. *Neurology International*.

[25] Penson D. F., Munro H. M., Signorello L. B., Blot W. J., Fowke J. H. (2011). Obesity, physical activity and lower urinary tract symptoms: results from the southern community cohort study. *Journal of Urology*.

[26] Kotsoris H., Barclay L. L., Kheyfets S., Hulyalkar A., Dougherty J. (1987). Urinary and gait disturbances as markers for early multi-infarct dementia. *Stroke*.

[27] Alexander M. S., Biering-Sorensen F., Bodner D. (2009). International standards to document remaining autonomic function after spinal cord injury. *Spinal Cord*.

[28] Liao W.-C., Jaw F.-S. (2010). A noninvasive evaluation of autonomic nervous system dysfunction in women with an overactive bladder. *International Journal of Gynecology and Obstetrics*.

[29] Umegaki H., Iimuro S., Shinozaki T. (2012). Risk factors associated with cognitive decline in the elderly with type 2 diabetes: baseline data analysis of the Japanese elderly diabetes intervention trial. *Geriatrics and Gerontology International*.

[30] Cersosimo M. G., Benarroch E. E., Ruud M. B., Dick F. S. (2013). Central control of autonomic function and involvement in neurodegenerative disorders. *Handbook of Clinical Neurology*.

[31] Benarroch E. E., Schmeichel A. M., Low P. A., Boeve B. F., Sandroni P., Parisi J. E. (2005). Involvement of medullary regions controlling sympathetic output in Lewy body disease. *Brain*.

[32] Braak H., Braak E. (1995). Staging of Alzheimer's disease-related neurofibrillary changes. *Neurobiology of Aging*.

[33] Collins O., Dillon S., Finucane C., Lawlor B., Kenny R. A. (2012). Parasympathetic autonomic dysfunction is common in mild cognitive impairment. *Neurobiology of Aging*.

[34] Pagoria D., O'Connor R. C., Guralnick M. L. (2011). Antimuscarinic drugs: review of the cognitive impact when used to treat overactive bladder in elderly patients. *Current Urology Reports*.

[35] Han L., Agostini J. V., Allore H. G. (2008). Cumulative anticholinergic exposure is associated with poor memory and executive function in older men. *Journal of the American Geriatrics Society*.

[36] Todorova A., Vonderheid-Guth B., Dimpfel W. (2001). Effects of tolterodine, trospium chloride, and oxybutynin on the central nervous system. *The Journal of Clinical Pharmacology*.

[37] Thor K. B., Kirby M., Viktrup L. (2007). Serotonin and noradrenaline involvement in urinary incontinence, depression and pain: scientific basis for overlapping clinical efficacy from a single drug, duloxetine. *International Journal of Clinical Practice*.

[38] Pietrzak R. H., Maruff P., Woodward M. (2012). Mild worry symptoms predict decline in learning and memory in healthy older adults: a 2-year prospective cohort study. *The American Journal of Geriatric Psychiatry*.

[39] Pietrzak R. H., Scott J. C., Neumeister A. (2014). Anxiety symptoms, cerebral amyloid burden and memory decline in healthy older adults without dementia: 3-year prospective cohort study. *British Journal of Psychiatry*.

[40] Geda Y. E., Roberts R. O., Mielke M. M. (2014). Baseline neuropsychiatric symptoms and the risk of incident mild cognitive impairment: a population-based study. *The American Journal of Psychiatry*.

[41] Bureau of National Health Insurance (2012). *National Health Insurance Annual Statistical Report 2011*.

